# Fast Pure Shift NMR Spectroscopy Using Attention‐Assisted Deep Neural Network

**DOI:** 10.1002/advs.202309810

**Published:** 2024-06-05

**Authors:** Haolin Zhan, Jiawei Liu, Qiyuan Fang, Xinyu Chen, Yang Ni, Lingling Zhou

**Affiliations:** ^1^ Department of Biomedical Engineering Anhui Provincial Engineering Research Center of Semiconductor Inspection Technology and Instrument Anhui Province Key Laboratory of Measuring Theory and Precision Instrument School of Instrument Science and Opto‐electronics Engineering Hefei University of Technology Hefei 230009 China

**Keywords:** artificial Intelligence, attention mechanism, deep learning, NMR spectroscopy, pure shift

## Abstract

Pure shift NMR spectroscopy enables the robust probing on molecular structure and dynamics, benefiting from great resolution enhancements. Despite extensive application landscapes in various branches of chemistry, the long experimental times induced by the additional time dimension generally hinder its further developments and practical deployments, especially for multi‐dimensional pure shift NMR. Herein, this study proposes and implements the fast, reliable, and robust reconstruction for accelerated pure shift NMR spectroscopy with lightweight attention‐assisted deep neural network. This deep learning protocol allows one to regain high‐resolution signals and suppress undersampling artifacts, as well as furnish high‐fidelity signal intensities along with the accelerated pure shift acquisition, benefitting from the introduction of the attention mechanism to highlight the spectral feature and information of interest. Extensive results of simulated and experimental NMR data demonstrate that this attention‐assisted deep learning protocol enables the effective recovery of weak signals that are almost drown in the serious undersampling artifacts, and the distinction and recognition of close chemical shifts even though using merely 5.4% data, highlighting its huge potentials on fast pure shift NMR spectroscopy. As a result, this study affords a promising paradigm for the AI‐assisted NMR protocols toward broader applications in chemistry, biology, materials, and life sciences, and among others.

## Graphical abstract

An attention‐assisted deep neural network is described for highly accelerated pure shift NMR spectroscopy. It enables the faithful recovery of weak signals and the distinction and recognition of close chemical shifts, even though with merely 5.4% NUS data, thus giving a boost to broader NMR applications in various branches of chemistry.

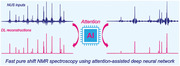

## 1 Introduction

Nuclear magnetic resonance (NMR) spectroscopy provides a powerful detection tool toward broader application scenarios in the fields of life science, chemistry, biology, and among others,^[^ ^1^ ^]^ benefiting from non‐invasively and quantitatively conveying atom‐scale structure and dynamic characteristics. 1D proton (^1^H) NMR that serves as the most sensitive and common detection technique among NMR spectroscopy families, nevertheless, is regrettably confined by the challenges of low spectral resolution and heavy signal overlap. Besides the classical multidimensional (mD) NMR experiments, recently, pure shift NMR techniques,^[^ ^2^ ^]^ which innovatively simplify the crowded multiplets induced by the extensive scalar coupling splittings into concise pure shift singlets, have been regarded as an alternative manner to address the resolution challenge and facilitate the spectral analysis. Also, combining the advantages of both techniques, 2D and mD pure shift NMR techniques have attracted more and more attention form chemists, and found extensive application landscapes in various branches of chemistry.^[^ ^3^ ^]^ In spite of the great resolution gains, lengthy experimental durations and low acquisition efficiency, induced by the additional time dimension for pure shift evolution except the real‐time ZS pure shift,^[^ ^2b^ ^]^ generally impose restrictions on their developments and practical deployments. Especially, this dilemma is further aggravated by the increasing transient scan numbers to accumulate signals of interest and remit the reduced spectral SNR, thus confining wider applications.

To address this, fast data acquisition is of great significance in pure shift NMR spectroscopy. The non‐uniform sampling (NUS)^[^ ^4^ ^]^ serves as a popular technique to enable fast acquisition and implement acceleration time benefits through reducing the amount of acquired data according to certain guidelines, thus improving the experimental efficiency. Due to the data missing, lots of artifacts are expectedly presented in the resulting spectra obtained by the Fourier transformation on the undersampled time‐domain free induced decay (FID) signals, rendering the extraction of desired spectral information challenging. Hence, faithful spectral reconstruction from undersampled signals is one of the frontiers and highly significant problems in the fields of chemistry, signal processing, and magnetic resonance.

To solve the underdetermined problem induced by the undersampling operator, various model‐based approaches, e.g., maximum entropy,^[^ ^5^ ^]^ compressed sensing (CS),^[^ ^6^ ^]^ spectral line shape estimation,^[^ ^7^ ^]^ and low‐rank Hankel reconstruction^[^ ^8^ ^]^ and so on, are established by introducing some specific priors, e.g., the low rankness of undersampled time‐domain signals and the sparsity of the resulting spectrum, to recover high‐fidelity NMR signals and implement high‐quality spectra. Among them, the state‐of‐the‐art compressed sensing approaches have witnessed the great success in fast biological NMR spectroscopy and beyond that. However, although these model‐based optimization algorithms provide the decent spectral reconstruction, a few conceptual questions and practical limitations still need to be addressed and improved, for example, lengthy computational times induced by a mass of iterative computations, inapplicability of certain assumptions of signal prior for some situations, as well as, manual parameter adjustments based on various situations.

Inspired by the prominent successes of artificial intelligence (AI) in various science and engineering fields, deep learning (DL)^[^ ^9^ ^]^ that automatically learns and implements feature mapping from a large training datasets between the given inputs and targeted results, has attracted increasing attention in enabling NMR spectroscopy.^[^ ^10^ ^]^ Thereinto, two convolutional neural network architectures, DLNMR^[^ ^10a^ ^]^ and FID‐Net^[^ ^10b^ ^]^ evolved from the conventional Dense^[^ ^11^ ^]^ and WaveNet^[^ ^12^ ^]^ networks, have been developed to recover 2D and mD biological NMR spectroscopy from NUS signals, from the insights of the frequency and time domains, respectively. Despite these successes, combined effects of insufficient generalization that generally require retraining for different application scenarios and tedious training durations (more than 10 h reported in theses literatures), somewhat limits these applications. In addition, although pure shift NMR has shown prominent achievements in various branches of chemistry, aforementioned deep neural network variants hardly focus on fast pure shift NMR spectroscopy, because pure shift NMR presents the conceptual and practical difference with conventional multidimensional NMR and certain pure shift chunks instead of points are omitted for NUS pure shift NMR. Very recently, the iterative compressed sensing algorithms^[^ ^13^ ^]^ and several successful DL practice,^[^ ^14^ ^]^ such as PS‐ResNet^[^ ^14a^ ^]^ modified from the original Res‐Net,^[^ ^15^ ^]^ have been introduced into pure shift NMR for specifically reconstructing undersampled pure shift NMR.

Herein, an attention‐assisted deep learning protocol for fast, reliable, and robust pure shift NMR spectroscopy reconstruction is implemented and validated. First, the deep learning protocol implements high‐quality NMR reconstruction by furnishing high‐resolution and artifact‐free spectra with high‐fidelity signal intensities form undersampled counterparts. Second, the proposed deep learning protocol introduces the attention mechanism to highlight the spectral feature and information of interest by automatically assigning different attentions according to the levels of interest on the spectral information, thus presenting satisfactory qualitative and quantitative performance and low reconstruction errors. Third, this lightweight DL proposal also adopts physics‐driven synthetic NMR data learning that lifts the prohibiting demand for a large volume of real training data generally required in DL practices, and implements the end‐to‐end feature learning that bypasses the iterative process and implements fast computation and reconstruction. Additionally, the trained 1D model is general for accelerated pure shift NMR spectroscopy on various chemical samples, and can be also exploited for multi‐dimensional pure shift NMR spectroscopy. Its performance has been adequately evaluated on simulated and experimental NMR spectra.

## 2 Experimental Section

### 2.1 Method

**Figure 1:**
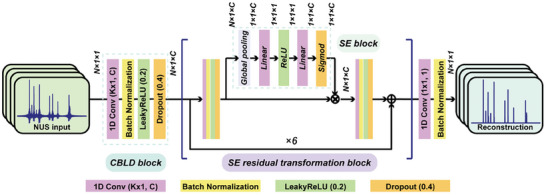
The architecture of SE‐PSNet for accelerated pure shift NMR spectroscopy. *N*x1 denotes the size of input data, and K and C are the kernel size and channel number, respectively. The original code scripts, trained models, as well as the example datasets are freely available at the online github website: https://github.com/HaolinZhan/SE‐PSNet.

As illustrated in **Figure** **1**, the main architecture of the proposed deep learning protocol for fast pure shift NMR, dubbed as SE‐PSNet (Squeeze‐and‐Excitation pure shift network), is composed of the CBLD block, and six SE residual transformation blocks, as well as the output block. First, the CBLD block composed of a convolution layer, batch normalization, LeakyReLU activation unit, and dropout operations was adopted to preliminarily extract desired features. Second, the SE residual transformation blocks adopted two CBLD blocks into the residual block, and accommodate the squeeze‐and‐excitation (SE) channel attention block^[^ ^16^ ^]^ in which the global pooling, the fully connectedly layer, ReLU activation unit, another fully connectedly layer, as well as sigmod activation unit were sequentially executed to adaptively learn the attention weights between different channels, in the middle of two CBLD blocks. Finally, the output block was constituted by a 1 × 1 convolution layer and batch normalization, to combine the feature information of multiple channels and afford the targeted high‐quality pure shift reconstruction results. The sizes of the feature mappings during the network learning are shown in the Figure 1. Removing the SE block, the architecture was named as the non‐SE‐PSNet, which held the simliar architecture to the PS‐ResNet^[^ ^14a^ ^]^ but removed the activation function and dropout layer in the output block for a better performance on original output intensity and peak resolution, as well as presented the difference on the kernel size and the number of the residual blocks.

The SE‐PSNet directly recovered high‐quality NMR spectra in the frequency domain instead of the time domain, to implement the bigger receptive field and better retain global information. Also, this deep neural network adopted the 1D rather that 2D convolution to afford the smaller model size and fewer weight parameters, highlighting the lightweight network advantages and boosting the potential applications on portable devices. In addition, the optimized convolution kernel size (namely 9 and 21 in CBLD and SE residual blocks, respectively) was adopted to optimize the receptive fields and improve the feature extraction capability for generally sparse NMR spectra. Then, the residual learning framework was introduced to bypass possible gradient vanishing issues. Noteworthily, the squeeze‐and‐excitation (SE) module was accommodated as an attention gate to adaptively learn the attention weights and capture the degree of importance of different channels, as well as to automatically assign different attentions according to the levels of interest on the spectral information. Combining the CNN and the attention mechanism, the SE‐PSNet paid more attention on spectral information of interest, and take full advantages of global and local features, thus enhancing the robustness in qualitative and quantitative performance in fast pure shift NMR spectroscopy. Besides, the size of the feature map remained consistent during the feature extraction and learning processes to avoid the information loss. Moreover, the adopted SE block hardly increased additional parameters and computational costs, benefitting from the data size of 1×1×1 during two linear layers. Furthermore, it had been also explored that the performance improvements produced by the SE block were fairly robust to its integration location into the residual block, provided that they were applied prior to branch aggregation, resembling that reported in the previous literature.^[^ ^16^ ^]^

Since enormous experimental NMR spectra were hardly accessible as a result of the limitations of NMR samples and instrument times, the proposed SE‐PSNet provides a compact protocol adopting physics‐driven synthetic data learning to optimize the feature map, similar to the successful practices in fast biological NMR spectroscopy.^[^ ^17^ ^]^ The synthetic datasets used for training and validation were generated following the physical formulas of pseudo‐2D pure shift NMR platforms (The schematic diagram of data chunk concatenation is shown in Figure S1, Supporting Information), in which merely the real parts of the simulated spectra were used to train the neural network, and no experimental NMR data was required, thus liberating the requirements on a huge volume of actual experimental data and beyond most traditional DL approaches. In addition, benefitting from the robust performance on feature learning, the SE‐PSNet usually required only a small amount of training samples, namely thousands of synthetic data, to attain satisfactory reconstructions, superior to competing DL proposals on fast biological NMR spectroscopy. As a result, the SE‐PSNet was trained to end‐to‐end learn the feature mapping from the NUS NMR signals to targeted high‐resolution artifact‐free noiseless pure shift spectra, where the ideal fully sampled spectra serve as the supervised labels during the training processes.

### 2.2 Experiments

Fully sampled pure shift NMR spectra of two common sample of 50mM quinine in CDCl_3_, β‐estradiol in CD_3_OD, and α‐pinene in CDCl_3_ adopted in the previous literature,^[^ ^4b^ ^]^ were downloaded from the website of http://nmr.cent.uw.edu.pl/downloads/. Also, fully sampled pure shift NMR spectrum of a mixture of butanol and butyric acid in D_2_O was acquired using the interferogram‐mode PSYCHE pulse sequence on a 500 MHz commercial Varian NMR spectrometer (^1^H frequency 500 MHz). The detailed spectral parameters are summarized in Table S2 (Supporting Information). The normalized real parts of experimental spectra after phase corrections were adopted for desired pure shift reconstruction using well‐trained DL models.

## 3 Results and Discussion

**Figure 2:**
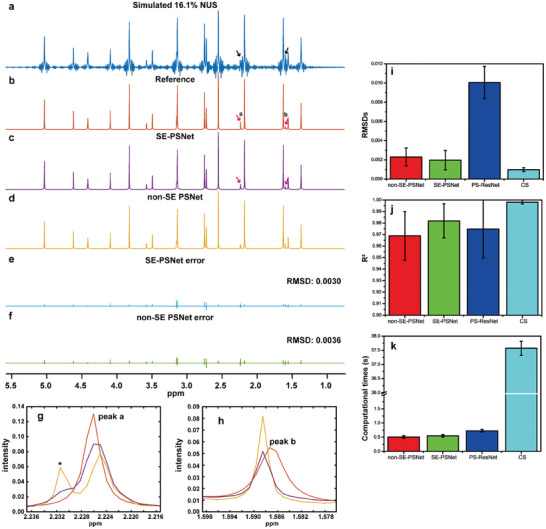
Accelerated pure shift NMR on simulation test data. a) 16.1% NUS pure shift spectrum. b) The ideal fully sampled pure shift counterpart as a reference. c,d) Reconstruction results of the SE‐PSNet with (SE‐PSNet) and without (non‐SE‐PSNet) the attention mechanism. e,f) Reconstruction errors between the normalized reconstructed and reference spectra of SE‐PSNet and non‐SE‐PSNet. g,h) The zooms of peaks **a** and **b** to demonstrate the detailed comparison, in which the red, purple, and yellow denote the reference, SE‐PSNet and non‐SE‐PSNet reconstruction results, respectively, and the asterisk (*) denotes the residual artifacts for the non‐SE‐PSNet reconstruction. i–k) The bar graphs indicate RMSDs, *R* ^2^, and computational times recorded over 20 random trials, and the heights of bar charts and error bars denote the averages and standard deviations, in which the RMSD and R^2^ values are calculated on network outputs. *R* ^2^ denotes the square of the Pearson correlation coefficient. The reconstruction times are counted on the indential CPU hardware.

To illustrate the potential of the proposed SE‐PSNet on accelerated pure shift NMR spectroscopy, the qualitative and quantitive performance have been validated on simulated and experimental NMR spectra. **Figure** **2** shows a satisfactory reconstruction results on a simulated data set with a NUS level of 16.1%, namely with an acceleration factor of 6.2. In this case where the weak signals adjacent to strong counterparts, namely peaks **a** and **b** marked by the arrows, are almost completely submerged in undersampling artifacts in 16.1% NUS spectrum (Figure 2a), the SE‐PSNet still enables their fast recovery and identification (see red arrow in Figure 2c) with a low reconstruction error illustrated in Figure 2e, suggesting high fidelities on signal intensities and reconstructed chemical shifts. In addition, compared to the ablation version without adopting attention mechanism (Figure 2d,f), the introduction of attention block (Figure 2c,e) contributes to the qualitative and quantitative performance enhancement and the reconstruction error reduction by ≈17%. Additionally, as shown in expanded small peaks in Figure 2g,h, the SE‐PSNet provides more faithful intensity reconstruction on weak signals and delivers the cleaner spectrum with the fewer residual artifacts. Besides, root‐mean‐squared deviations (RMSDs) between the fully sampled and reconstructed spectra, peak intensity correlation *R* ^2^, as well as the reconstruction times have been adopted for a quantitative comparison between the SE‐PSNet with and without the attention block, a start‐of‐art DL protocol PS‐ResNet,^[^ ^14a^ ^]^ as well as one of classical CS algorithm (here IST‐S^[^ ^6a^ ^]^), over 20 random simulated samples. And the statistical results (Figure 2i‐k) demonstrate that among the DL protocols, the proposed SE‐PSNet provides a lower RMSDs and a higher peak intensity correlation *R* ^2^, suggesting the robust reconstruction performance. Furthermore, resulting from the iterative computational procedure, the conventional CS algorithms delivers the superior quantitative results for a higher NUS level, such as 16.1% NUS, especially on simulated data, despite suffering from the possible baseline distortions, but also requires the more computational times by about two orders of magnitude that may limit broader applications on large data sizes and multidimensional NMR data.

**Figure 3:**
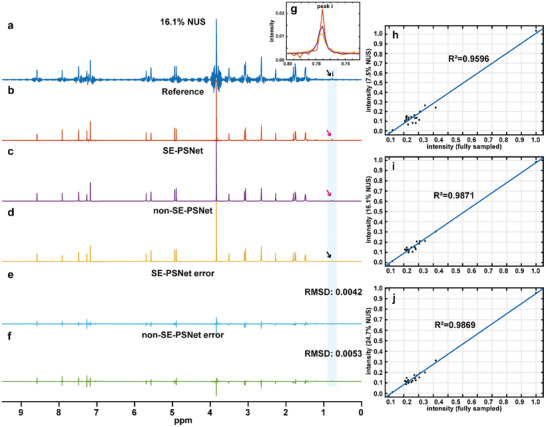
Accelerated pure shift NMR on quinine. a) 16.1% NUS pure shift spectrum. b) The fully sampled pure shift counterpart as a reference. c,d) Reconstruction results of the SE‐PSNet (c) and non‐SE‐PSNet (d). e,f) Related reconstruction errors between the normalized reconstructed and reference spectra. g) The zoom of small peak **i** marked by the arrow to demonstrate more faithful weak peak reconstruction, in which the red, purple, and yellow denote the reference, SE‐PSNet recontruction and non‐SE‐PSNet reconstruction results, respectively. h–j) Quantitative evaluation on peak intensity correlations between the fully sampled spectrum and the reconstructed spectrum of three NUS levels, namely 7.5% NUS (g), 16.1% NUS (h), 24.7% NUS (i).

**Figure** **3** illustrates a practical example on accelerated pure shift NMR of quinine, a clinical drug for malaria exhibiting complicated molecular structure and crowded spectral distribution, in which pure shift spectroscopy plays an important role in resolving its spectra and then disentangling its structure. Because merely 16.1% pure shift data chunks are adopted, severe spectral artifacts and inevitable resolution degradations appear in the resulting NUS spectrum (Figure 3a), thus confusing actual peaks with spurious signals. Referring to the fully sampled pure shift counterpart (Figure 3b), the SE‐PSNet (Figure 3c) allows one to recover the high resolution, suppress all spectral artifacts including undersampling artifacts and chunk sidebands, retain desired peaks with no introduction of false positive signals. Notably, the SE‐PSNet remains efficient for peak intensities with high dynamic range, such as in this case the highest intensity of the spectral peak approaches 50 times of the lowest intensity one, namely peak **i**, which is associated with the quinine photodegradation,^[^ ^18^ ^]^ raising the challenging reconstruction of weak peaks. Compared to the non‐SE‐PSNet (Figure 3d) without the attention mechanism, the SE‐PSNet furnishes the higher‐fidelity reconstruction on peak intensities and frequencies, as shown in Figure 3g, especially for the weak peak **i** whose intensity is obviously lower than the undersampling artifacts. Regarding the quantitative evaluation, Figure 3e,f shows that the reconstruction error of the SE‐PSNet is merely 79.2% of the non‐SE‐PSNet. Additionally, three NUS levels, namely 7.5%, 16.1%, and 24.7% are also tested to certify the robust reconstruction performance of the SE‐PSNet, and quantitative evaluations (Figure 3h–j) validate its robust performance. Besides, the reconstruction performance with different NUS levels has been also investigated on a simulated sample and practical quinine sample, and the results are given in Figures S3 and S4j (Supporting Information). It can been seen that for 5.4% NUS, the peak intensity correlation R^2^ values, are 0.8661 and 0.8627 on the simulated sample (Figure S3, Supporting Information) and quinine sample (Figure S4, Supporting Information), respectively. Along with the increasing NUS levels, the quantitative performance regarding peak intensity correlations (R^2^) and root‐mean‐squared deviations (RMSDs) gradually improves, as shown in Figures S3d and Figure S4j (Supporting Information). For 16.1% NUS, the R^2^ values reach 0.9946 and 0.9871, suggesting the high‐fidelity reconstruction on peak intensity, while 0.9952 and 0.9869 for 24.7% NUS. Thus, with a compromise with the acceleration benefit and reconstruction fidelity, the 16.1% NUS is chosen to demonstrate the reconstruction performance with an acceleration factor of 6.2. Moreover, Figure S5 (Supporting Information) also presents similar reconstruction results on another chemical samples, β‐estradiol that is also characterized with complicated molecular structure. Moreover, as summarized in **Table** **1**, the SE‐PSNet presents the advantages of lightweight network, and holds the short model training times (≈20 min) and the small model size (≈1 MB), as well as fast reconstruction times (0.57 s on CPU). As a result, the proposed lightweight neural network SE‐PSNet constitutes a powerful tool for fast, high‐quality, and reliable pure shift NMR acceleration.

**Table 1:** Parameter comparison between different methods for pure shift reconstruction.

Methods^a^ ^)^	Required model training times (s)^b^ ^)^	Model sizes	FLOPs (billion)^d^ ^)^	Reconstruction time (s)^e^ ^)^
SE‐PSNet	1323	1.03 MB	2.13	0.57
Non‐SE PSNet	1303	1.02 MB	2.13	0.52
PS‐ResNet^[^ ^14a^ ^]^	1522	215.45 KB/1.62 MB^c^ ^)^	3.38	0.72
1D U‐Net^[^ ^19^ ^]^	2699	102.79 MB	—	3.12
IST‐S^[^ ^6a^ ^]^	——			40.43

^a)^ The identical training set was adopted and the model training were carried out on the same computing device. The detailed network parameters and model training processes are given in the Supporting Information;

^b)^ The network training is performed on a server (Intel Core i9‐10900X CPU@ 4.50 GHz and 128 GB RAM) equipped with an GeForce RTX 3080 Ti GPU;

^c)^ Two PS‐ResNet models are trained using different styles of code writings of network structure, and the model size reaches 1.62 MB when the style of code writings is unified as those of the other methods;

^d)^ FLOPs denotes floating point operations (multiply‐adds), which indicate the computation complexity;

^e)^ The reconstruction is performed on the identical personal computer using the CPU (Intel Pentium Gold G5400).

**Figure 4:**
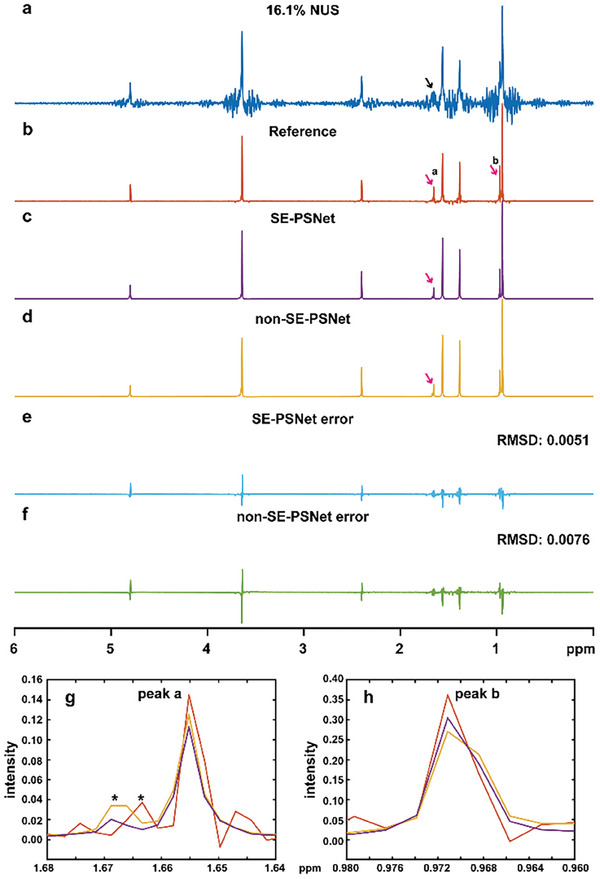
Accelerated pure shift NMR on a mixture of butanol and butyric acid. a) 16.1% NUS pure shift spectrum. b) The fully sampled pure shift counterpart as a reference. c,d) Reconstruction results of the SE‐PSNet with and without the attention mechanism. e,f) Related reconstruction errors between the normalized reconstructed and reference spectra. g,h) The zooms of peaks a and b, in which the red, purple, and yellow denote the reference, SE‐PSNet and non‐SE‐PSNet reconstruction results, respectively, and the asterisk (*) denotes the residual artifacts.

To further demonstrate the applicability and powerfulness of the proposed SE‐PSNet on mixture systems with crowded resonances and large dynamic ranges, a mixture containing two components, butanol and butyric acid, whose concentrations exhibit a five‐fold difference, is tested. As shown in **Figure** **4**a, it is observed that the severe undersampling artifacts completely submerge the peak **a** belonging to the diluted component in 16.1% NUS spectrum, referred to the fully sampled pure shift spectrum (Figure 4b). Fortunately, the proposed DL protocol (Figure 4c) enables the recovery of all desired peaks including peak **a**. In addition, in contrast to the non‐SE‐PSNet (Figure 4d), the SE‐PSNet introduces fewer false positive and false negative peaks, and provides more faithful peak intensities, as shown in Figure 4g,h. Besides, as depicted in Figure 4e,f, the reconstruction error of SE‐PSNet is merely 67% that of non‐SE‐PSNet, further declaring the powerfulness of attention mechanism for pure shift reconstruction.

**Figure 5:**
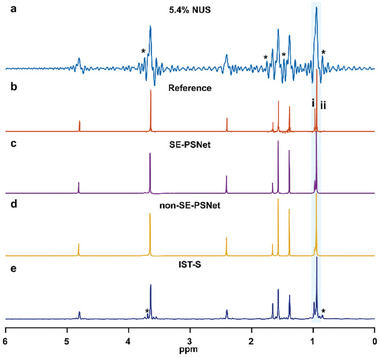
Accelerated pure shift NMR on a mixture of butanol and butyric acid. a) 5.4% NUS pure shift spectrum. b) The referenced fully sampled pure shift spectrum that is the same as that in Figure 4b. (c‐e) Reconstruction results of the SE‐PSNet (c), non‐SE‐PSNet (d), and classical IST‐S algorithm (e). For the IST‐S reconstruction, 500 iterations and the threshold value of 0.99 are adopted. The asterisks (*) indicate the undersampling artifacts whose intensities approach or exceed the desired actual signals.

Another extreme example is illustrated on the reconstruction on mixture of butanol and butyric acid with merely 5.4% NMR data. Obviously, it is observed that a number of undersampling artifacts marked with the asterisks, whose intensities approach even exceed the desired actual signals, inevitably appear in the resulting NUS spectrum (**Figure** **5**a), and a broadening peak envelope almost cover the peaks **i** and **ii** with close chemical shifts, along with the extremely low sampling density, thus hindering the spectral analysis. By contrast, the proposed SE‐PSNet (Figure 5c) remains capable of affording high‐resolution pure shift spectrum, in which all peaks are identified and originally intertwined peaks **i** and **ii** are clearly distinguished, indicating a prominent time benefit of about a factor of 20 for fast pure shift NMR spectroscopy. Nevertheless, it should be honestly noted that along with the extremely low sample density of 5.4%, the reconstruction performance on signal intensities, i. e., peak **i**, is somewhat degraded, despite with a decent signal resolution. Moreover, the SE‐PSNet still delivers the superior reconstruction results compared to the non‐SE‐PSNet, in terms of both signal resolution gain and signal strength fidelity. In addition, for 5.4% NUS, resulting IST‐S spectrum (Figure 5e) suffers from a few artifacts marked by the asterisks (*), thus reducing the reconstruction quality and challenging the extraction of the spectral information. As a consequence, this presented DL protocol enables the high‐quality pure shift reconstruction with very little NMR data, suggesting huge potentials on fast pure shift NMR spectroscopy.

### 3.1 Discussion

Apart from the aforementioned examples using the intensities, namely peak heights, to evaluate the quantitative performance, the quantitative evaluation using integrals is also performed on quinine sample. As shown in Figure S6 (Supporting Information), the results still verify the satisfactory quantitative performances with superior R^2^ values than those using intensities originally shown in Figure 3. Apart from R^2^, the RMSD values between the reconstruction results and label regarding the peak regions are also calculated, and the higher R^2^ and smaller RMSDs than the non‐SE‐PSNet with 16.1% NUS also explain its superior quantitative performance of the SE‐PSNet with three NUS levels. In addition, the reconstruction performance in the presence of noise is also evaluated, and reconstruction results on quinine and β‐estradiol with different noise levels are given in Figures S7 and S8 (Supporting Information). As a result, the proposed SE‐PSNet allows one to reconstruct high‐quality pure shift spectra even though in presence of a typical amount of noise, and still enables the identification and recovery of the weak signals almost completely drown in the noisy fully sampled and NUS spectra. Furthermore, a larger size of the datasets, namely 80k, is also adopted for training and validating to verify the feasibility of the proposed SE‐PSNet with a small amount of synthetic sample learning, here several thousands. It can be seen in Figure S9 (Supporting Information) that although the number of the training datasets is notably increased to 80k from the original 4k, the performance improvement is hardly observable, thus excluding the overfitting possibility of the SE‐PSNet models, and the smaller deviations of the SE‐PSNet also suggest its more robust and reliable performance by the incorporation of SE block, compared to the non‐SE‐PSNet. Moreover, the SE‐PSNet reconstruction results adopting a larger Fourier transformation (FT) point of 16384 instead of the original 8192 are also given in Figure S10 (Supporting Information) to deliver the more smoothed lines.

Classic iterative algorithms and deep learning proposals have been regarded as two state‐of‐the‐art techniques for reconstructing NUS NMR spectroscopy, and have witnessed some degree of success in various fields. And the performance comparison between the proposed SE‐PSNet and other state‐of‐the‐art deep learning and compressed sensing approaches is also shown in Figure 2i–k and Table 1 in details. By constrast of the iterative IST‐S method,^[^ ^6a^ ^]^ one of compressed sensing methods, the DL protocols hardly request the time‐consuming and subjective parameter optimization, and enable very fast reconstructions by nearly two orders of magnitude more than the iterative methods, benefitting from the non‐iterative low‐complexity feature mappings. Among the DL protocols, it is demonstrated that the proposed SE‐PSNet delivers robust performance on accelerated pure shift NMR, in terms of both signal resolution gain and signal intensity fidelity, exceeding the competitors, PS‐ResNet^[^ ^14a^ ^]^ and 1D U‐Net.^[^ ^19^ ^]^ A detailed comparison among the reconstruction performance among original PS‐ResNet, non‐SE‐PSNet, and SE‐PSNet, is also illustrated in Figure S11 (Supporting Information), verifying the powerfulness of the proposed SE‐PSNet and the necessity of the attention mechanism block. In addition, the experimental comparison between the proposed SE‐PSNet and the recent DL‐PSNMR^[^ ^14b^ ^]^ designed on aggregated residual transformation is performed on 5.4% NUS α‐pinene and β‐estradiol, and reconstruction results (Figure S12 and S13) verify the advantages of the SE‐PSNet over DL‐PSNMR. Besides, as a lightweight neural network, the SE‐PSNet protocol remains superior to existing DNN‐based methods, i. e., DL NMR,^[^ ^10a^ ^]^ FID‐Net,^[^ ^10b^ ^]^ DHMF,^[^ ^20^ ^]^ MoDern,^[^ ^21^ ^]^ which have been powerful in NMR acceleration and reconstruction, benefitting from the few required amounts of training datasets, the short model training times, the small model size, as well as the fast computational efficiency. Moreover, this study provides a new perspective for DL‐based NMR spectroscopy reconstruction by introducing the concept of attention‐assisted deep neural network for accelerated pure shift NMR spectroscopy. As reasonable extensions, not only the demonstrated SE blocks but also other attention mechanism blocks, such as the improved CBAM^[^ ^22^ ^]^ and the Transformer,^[^ ^23^ ^]^ may also hold potentials on enabling NMR spectroscopy according to practical application scenarios. Also, the attention mechanism is also expected to be applicable to address other issues in NMR fields.

As demonstrated in aforementioned examples, the SE‐PSNet protocol serves as one of powerful tools for fast, reliable, and robust reconstruction on pure shift NMR spectroscopy. Despite these gains, its limitations on further applications still need to be discussed. First, since the DL proposals learn prior knowledges and desired features from the given datasets, the used training datasets unavoidably influence the reconstruction performance. Thus, training datasets considering multiple possibilities, such as chemical shift difference between peaks, noise levels, suitable signal dynamic intensity ranges, and so on, are designed and generated aiming at required application scenarios. For example, a training dataset with bigger noise ranges may deliver decent reconstruction results on diluted samples. Additionally, in this paper, a trained network is applied to reconstruct data with a sampling rate. Provided that the training data sets deviate much in sizes of NUS density or exhibit different sampling schemes with the tested samples, the network retraining remains required. Nevertheless, the M‐to‐S SE‐PSNet (Figure S14, Supporting Information) adopting M‐to‐S strategy^[^ ^14^, ^24^ ^]^ that multiple undersampled spectra with different sampling schemes and NUS levels refer to the same ideal pure shift counterparts, has been also proven to be helpful to enhance the generalization ability of DL protocols. As a result, provided that the model is trained based on the ideal training datasets fully considering multiple possibilities, the SE‐PSNet may hold the decent potential on various chemical samples and NMR platforms. In addition, the trained 1D SE‐PSNet model may be also applicable to multi‐dimensional pure shift NMR by line‐to‐line reconstruction. Second, the current DL methods remain incapable to deal with the extreme cases that serious peak broadening effects and undersampling artifacts completely convert the peaks with close chemical shifts into a broad envelope, and that the desired NMR signals are below the detection limit of the instruments. Even though, the SE‐PSNet has demonstrated somewhat the robust performance on recovering the weak signals, for example peak **i** in Figure 3c whose intensity is merely 2.2% of the highest peak intensity and approaches even is lower than the undersampling artifacts, and distinguishing close chemical shifts, e. g., peak **i** and **ii** for 5.4% NUS in Figure 5c. Third, due to the end‐to‐end training pattern, common to generic deep networks, the proposed DL scheme is inevitably confined by a lack of interpretability. To address it, the deep networks designed by iterative algorithm unrolling^[^ ^25^ ^]^ may be a solution, which is also actively exploring. Moreover, it should be noticed that there still exists a distance to bridge the gap between the DL protocols and practical applications, despite hopefulness.

## 4 Conclusion

In conclusion, we propose and implement the attention‐assisted deep learning protocol, for robust, reliable, and ultrafast pure shift NMR spectroscopy reconstruction. Based on the introduction of attention mechanism, the presented DL protocol allows one to regain high‐resolution signals and suppress undersampling artifacts, as well as furnish high‐fidelity signal intensities form NUS pure shift acquisition, even though using merely 5.4% NUS data. Additionally, it also benefits to the lightweight advantages of the rapid model trainings, the small model sizes, fewer weight parameters, as well as fast computational efficiency with a small amount of physics‐driven synthetic NMR spectra learning, thus somewhat liberating specialized requirements on advanced computing hardwares. Furthermore, this protocol is general to diverse chemical and biological samples, and also potential to multi‐dimensional pure shift spectroscopy via a line‐by‐line reconstruction. Benefitting from the highly accelerated pure shift implementation for ultrahigh‐resolution access to NMR peaks, the proposed SE‐PSNet protocol may find potential application landscapes in fast disentangling molecular structure and dynamics information for molecules with complex chemical structure, especially for low‐field portable NMR detections. As a consequence, this study provides an effective DL methodology for enabling NMR spectroscopy acceleration, and takes a meaningful step for bridging the gap between DL protocols and AI‐assisted chemistry.

## Conflict of Interest

The authors declare no conflict of interest.

## Supporting information

Supporting Information

## Data Availability

The data that support the findings of this study are available from the corresponding author upon reasonable request.
