# Supplementary material for: Fast Pure Shift NMR Spectroscopy Using Attention‐Assisted Deep Neural Network
Source: Adv Sci (Weinh). 2024 Jun 5;11(29):2309810. doi: 10.1002/advs.202309810 (PMC11304274; doi:10.1002/advs.202309810)
Supplement: Supplementary file 1 — Supporting Information [file ADVS-11-2309810-s001.docx]

Supporting Information
©Wiley-VCH 2016
69451 Weinheim, Germany

Fast pure shift NMR spectroscopy using attention-assisted deep neural network

Haolin Zhan^*^, Jiawei Liu, Qiyuan Fang, Xinyu Chen, Yang Ni, Lingling Zhou

**E-mail: hlzhan@hfut.edu.cn**

**Abstract:** Pure shift NMR spectroscopy enables the robust probing on molecular structure and dynamics, benefiting from great resolution enhancements. Despite extensive application landscapes in various branches of chemistry, the long experimental times induced by the additional time dimension generally hinder its further developments and practical deployments, especially for multi-dimensional pure shift NMR. Herein, this study proposes and implements the fast, reliable, and robust reconstruction for accelerated pure shift NMR spectroscopy with lightweight attention-assisted deep neural network. This deep learning protocol allows one to regain high-resolution signals and suppress undersampling artifacts, as well as furnish high-fidelity signal intensities along with the accelerated pure shift acquisition, benefitting from the introduction of the attention mechanism to highlight the spectral feature and information of interest. Extensive results of simulated and experimental NMR data demonstrate that this attention-assisted deep learning protocol enables the effective recovery of weak signals which are almost drown in the serious undersampling artifacts, and the distinction and recognition of close chemical shifts even though using merely 5.4% data, highlighting its huge potentials on fast pure shift NMR spectroscopy. As a result, this study affords a promising paradigm for the AI-assisted NMR protocols toward broader applications in chemistry, biology, materials and life sciences, and among others.

DOI: 10.1002/anie.2023XXXXX

Training dataset generation and neural network training

Most pure shift techniques adopt the additional time dimension *t*_1_ for pure shift evolution, this is pseudo-2D pure shift NMR, apart from the real-time ZS. Provided that the design of homonuclear decoupling modules^[1]^ for pure shift evolutions, *J* couplings are theoretically refocused at the middle of data chunks of 1/SW1 duration. Then, a series of pure shift chunks with incremental time variables *t*_1_ are sequentially concatenated to reconstruct desired 1D pure shift spectroscopy. Thus, the signals acquired by the fully sampled pure shift spectra are written as follows:

$FID(t_{1},t_{2})=\sum_{p=1}^{P} [A_{p}e^{i2\pi f_{p}(t_{1}+t_{2})}e^{-(t_{1}+t_{2})/(T_{2})_{p}}\cos(\pi J_{p}(t_{2}-1/2SW_{1}))^{J_{-}num}],$ (S1)

in which *t*_2_ denotes a time variable runing from 0 to 1/SW1 during the chunks of 1/SW1 duration, and *P* is the total number of peaks in the generated spectra, *J*__num_ is the number of the coupled atoms, as well as *A_p_*, *f_p_*, (*T*_2_)*_p_,* and *J_p_*indicate the amplitudes, frequences, transverse relaxation times, and displayed coupling constants of each NMR peak *p*, respectively.


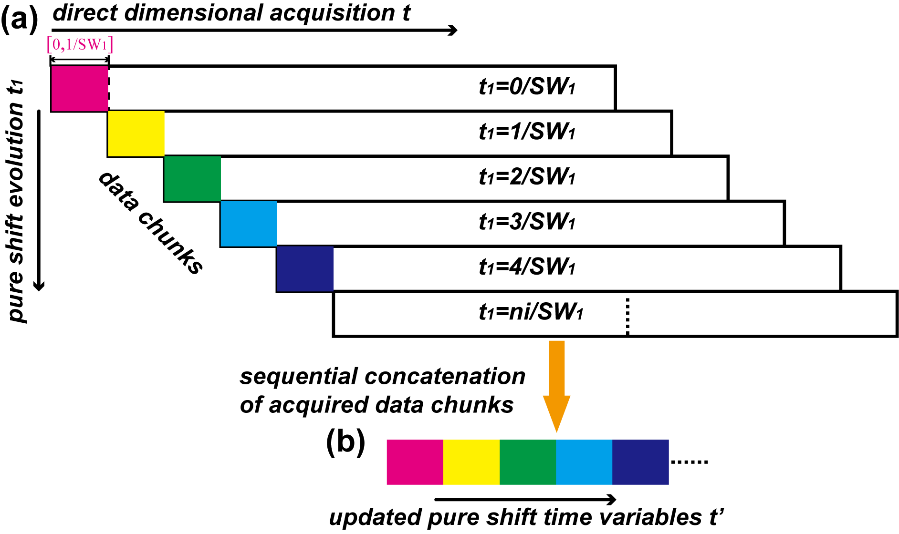


**Figure S1.** The schematic diagram of data chunk concatenation for pseudo-2D pure shift data chunking.

Then, NUS schedules, i. e., exponential sampling, Poisson sampling and burst sampling^[2]^, are executed to obtain the undersampled signals. Note that certain pure shift chunks instead of points are omitted and the points in the reserved chunks are generally continuous, different from common concept and practice in NMR undersampling. Also, the Gaussian white noises are added on the undersampled signals to enhance the robustness on possible noises in actual measurments.

$N\text{oise}=\alpha\times rand(1)\times(randn(np,1)+i\times randn(np,1)),$ (S2)

where *np* is the number of time-domain points of the fully sampled spectra, *α* denotes the standard deviation of noise levels adopting in training datasets. Thus, the pure shift signals with noise can be expressed as:

(S3)

Peak parameters are randomly selected for each plane in the training datasets to simulate various application scenarios, detailed parameters are summarized in the Table S1. As a result, the input data (***U***) of the training datasets are obtained *via* the Fourier transformation on the *FID_Noise*, as well as intensity normalization. By contrast, the ideal pure shift signals are generated by gathering a series of pure shift singlets as the supervised labels (***L***), following the formula described as follows:

(S4)

in which *t* denotes the time variables corresponding to *t*_1_+*t*_2_ in Eq. S1.

Given the synthetic undersampled spectrum (***U***) and the corresponding targeted ideal labels (***L***), a large number of data pairs, i. e., the *k*-th pair of (*U_k_*, *L_k_*) in which *k* is a integer variable running from 1 to the number of training datasets *K* (here *K* is 4000), are fed into the SE-PSNet to learn the optimal group of network parameters *θ*, *via* minimizing the mean absolute error (MAE) between the reconstruction results of the model *R_k_*, namely *R_k_*=*F(U_k_,θ)*, and corresponding labels *L_k_* in the frequency domain. The loss function is denoted as follows:

(S5)

While the network model is well trained, for a given undersampled spectrum *U*, a targeted high-quality pure shift counterpart *R* after the intensity normalization can be implemented *via* *R*=*F*(*U*, *θ*), and then exploited for fast pure shift NMR spectroscopy on random chemical samples, and also applicable to 2D and mD pure shift NMR by line-to-line reconstruction, indicating the robust generalization.

**Table S1. Parameters of synthetic NMR FID signals**

| Parameters | Value ranges |
| --- | --- |
| The number of peaks (P) | 7-26 |
| Amplitude (A) | 1-30 |
| Frequency range (f, Hz) | 1000-3000 |
| Coupling constants (J, Hz)  The number of coupling protons (*J_num_*) | 2-12  1 |
| Transverse relaxation time (T2, s) | 0.08-0.6 |
| Spectral width in the direct dimension (SW, Hz) | 4400 |
| Spectral width in the indirect dimension (SW1, Hz) | 50 |
| The number of pure shift data chunks | 93 |
| The number of sampling points in each chunk | 88 |
| The number of peaks (P) | 7-26 |

To evaluate the overall reconstruction errors and peak intensity fidelity for quantitative analysis, two indexes of root mean squared deviations (*RMSDs*) and the square of the Pearson correlation coefficient (*R*^2^) of each peak are calculated below:

(S6)

$R^{2}(PR,PL)=\text{（}\frac{\sum_{i=1}^{P} (PR_{i}-\overline{PR})(PL_{i}-\overline{PL})}{\sqrt{\sum_{i=1}^{P} (PR_{i}-\overline{PR})^{2}}\sqrt{\sum_{i=1}^{P} (PL_{i}-\overline{PL})^{2}}}\text{）.}$ (S7)

in which denotes the 2-norm of the error between the reconstruction results (*R*) and the corresponding labels (*L*), and *P* is the number of spectral peaks, as well as and are the mean values of peak intensities of the reconstruction (*PR*) and label spectra (*PR*). And peak intensities are measured by the peak heights.

Herein, 4000 ideal noise-free fully sampled pure shift signals following Eq. S4. Then, the white Gaussian noise with *α*=0.5 is added to each synthetic label. Generally, it was observed that the adopted noise level (namely the noise range of 0-0.5) is not obvious, and did not affect the DL NMR reconstruction since all pure shift NMR spectra, that presented in the main text and Supporting Information, were reconstructed well with the trained network under this noise level. And training datasets adopting a higher noise level may be applicable to dealing with the low NMR cases. Adpoting the parameters listed in the Table S1, 4000 pairs of undersampled and label spectra, namely (***U***, ***L***) are correspondingly generated, where the NUS pattern follows the exponential sampling scheme. Thus, 4000 synthetic data pairs were fed into the network for training, in which 80% namely 3200 pairs are used in training, and 20% namely 800 pairs that are excluded in training for validation. As shown in Figure S2, the decreasing loss uniformly presents the good convergence for the training and validation.


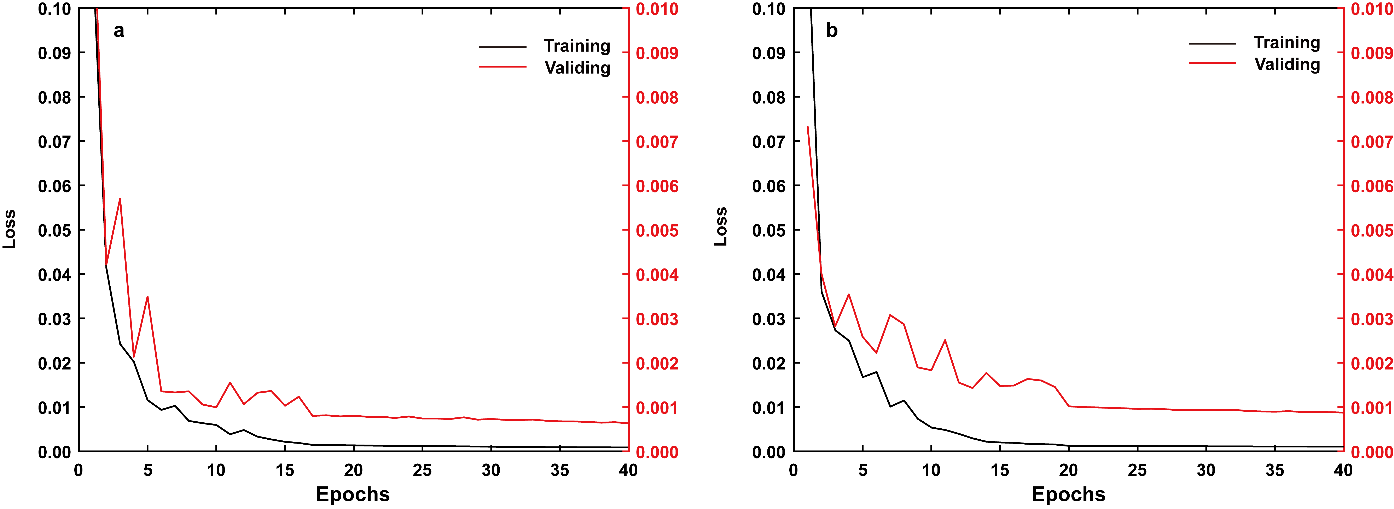


**Figure S2.** The training and validation loss curves of the SE-PSNet with 16.1 NUS (a) and 7.5% NUS (b). Both models adopts 4000 synthetic samples for network training (80%) and validation (20%).

The DL PSNMR models were trained on a server (Intel® Core™ i9-10900X CPU@ 4.50 GHz and 128 GB RAM) equipped with an GeForce RTX 3080 Ti GPU. The kernel sizes of the conventional layers in CBLD block and SE residual transformation block were set as 9 and 21 (K=9 and K=21), respectively. Additionally, a fixed feature channel number of 32 (C=32) was used with stride=1 and the paddings of the CBLD block and SE residual transformation block were set as 4 and 10 to ensure the consistent size of the feature map. The batch size is 32, and the weight initialization was not adopted. The initial learning rate is 10^-3^, and is reduced by a factor of 10 when the loss of validation set stops decreasing. Besides, the RMSprop optimizer was adopted to train the CNN neural network, and early stopping is used to determine the number of iterations (epochs).

Experimental NMR spectra

**Table S2. The number of sampling points and FT points of test samples**

| Parameters  Samples | Spectral width (*SW*) | Total data chunks | Time-domain points | Experimental times |
| --- | --- | --- | --- | --- |
| Quinine in CDCL_3_^a^ | 11160.7 Hz | 93 | 8112 | 13 mintues 10 seconds^b^ |
| β-estradiol in CD_3_OD^a^ | 11160.7 Hz | 93 | 8112 | 13 mintues 10 seconds^b^ |
| Butanol and butyric acid in D_2_O | 11160.7 Hz | 93 | 8112 | 13 mintues 19 seconds |

^a^ These spectral data were downloaded from the website of http://nmr.cent.uw.edu.pl/downloads/, which were also adopted in the previous literature^[2]^.

^b^ Experimental times were listed as the provious literature reported^[2]^.

Results and discussion


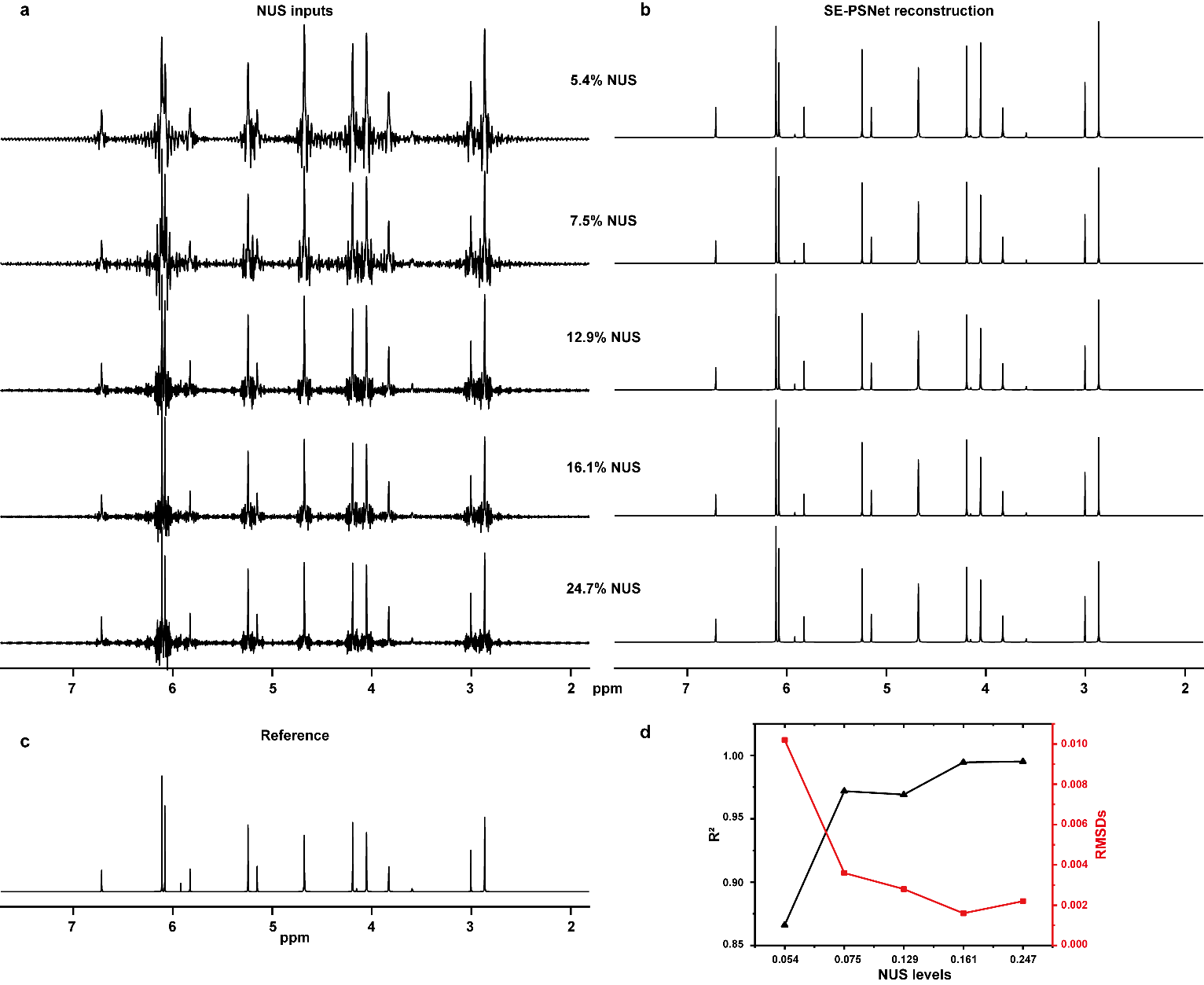


**Figure S3.** Accelerated pure shift NMR on another simulated sample with different NUS levels varying from 5.4% to 24.7%. (a, b) Five NUS inputs (a) and related SE-PSNet reconstruction results (b) of 5.4 %, 7.5%, 12.9%, 16.1% and 24.7%. (c) The ideal pure shift spectrum as the reference. (d) Quantitative evaluation on R^2^ and RMSDs between the normalized reconstructed and reference spectra *vs* five NUS levels.


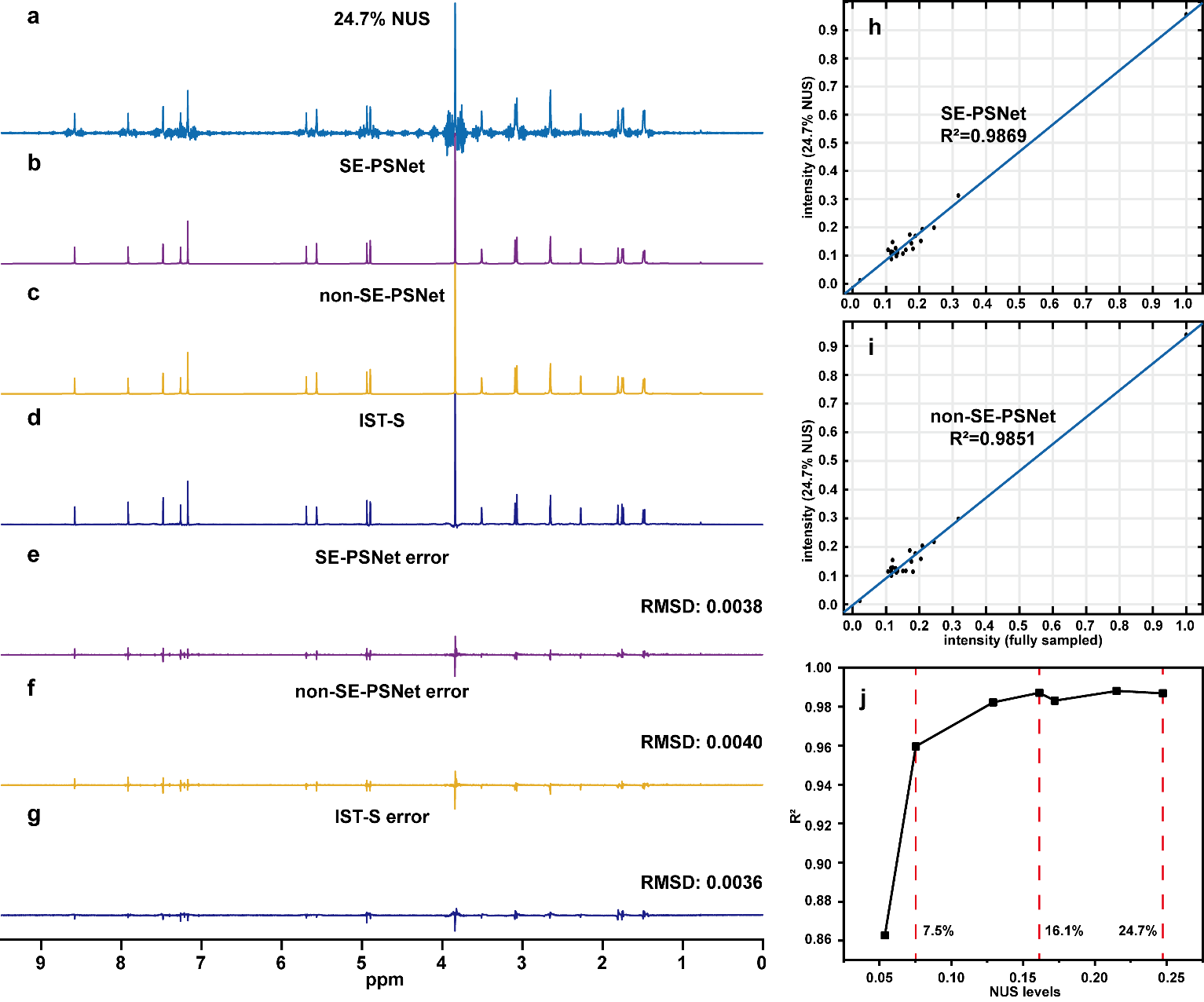


**Figure S4.** Pure shift NMR reconstruction on quinine. (a) 24.7% NUS pure shift spectrum. (b-d) Reconstruction results of the SE-PSNet (b), non-SE-PSNet (c), as well as the classical IST-S algorithm (d). (e-g) Reconstruction errors between the normalized reconstructed and reference spectra of SE-PSNet (e), non-SE-PSNet (f), and IST-S (g). (i, j) Quantitative evaluation on peak intensity correlations of the SE-PSNet (h) and non-SE-PSNet (i). (j) Quantitative R^2^ values on different NUS levels.


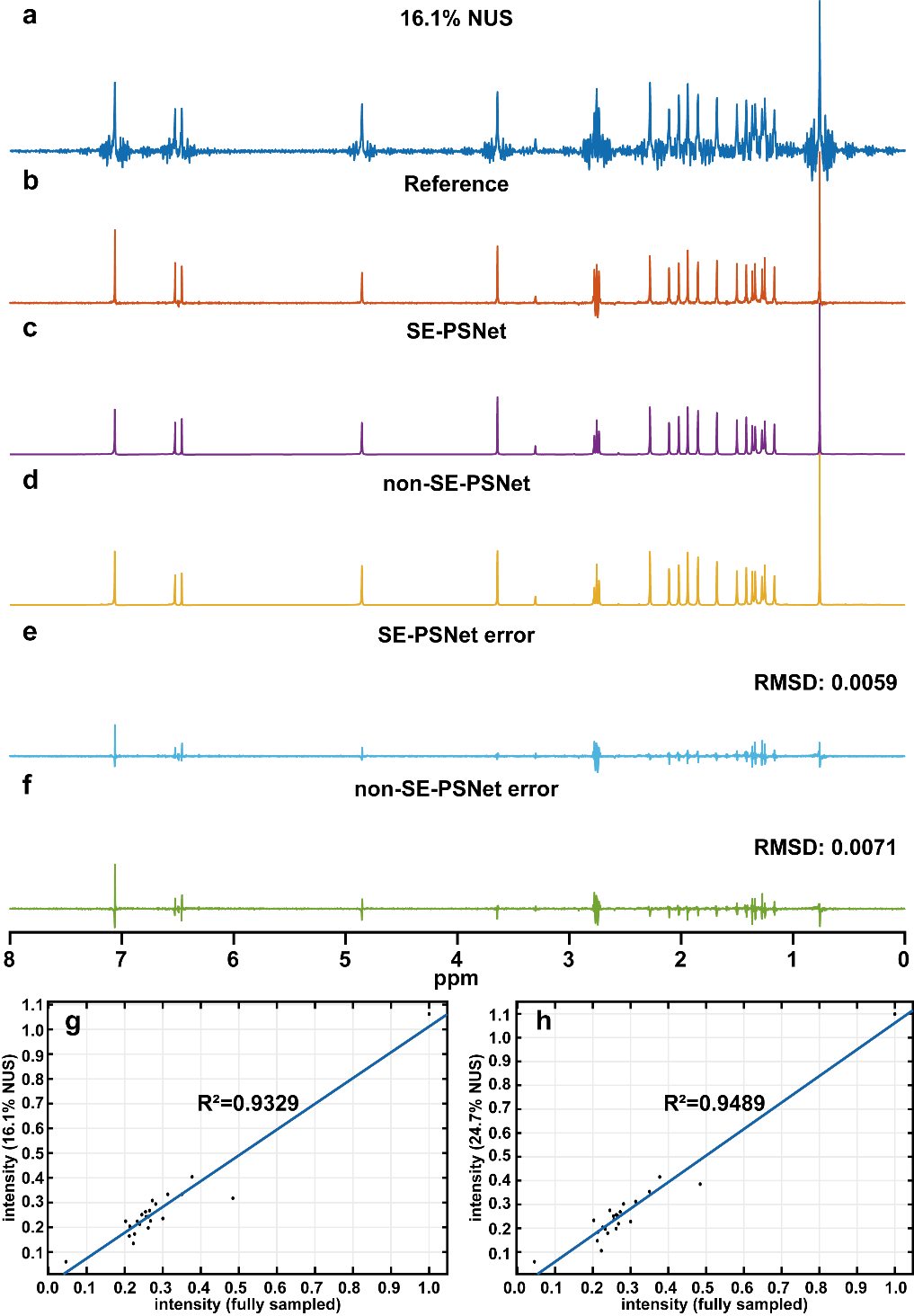


**Figure S5.** Accelerated pure shift NMR on β-estradiol. (a) 16.1% NUS pure shift spectrum. (b) The fully sampled pure shift counterpart as a reference. (c, d) Reconstruction results of the SE-PSNet with and without the attention mechanism. (e, f) Reconstruction errors between the normalized reconstructed and reference spectra of SE-PSNet and non-SE-PSNet. (g-h) Quantitative evaluation on peak intensity correlations between the fully sampled spectrum and the reconstructed spectrum of two NUS levels of 16.1% NUS (g) and 24.7% NUS (h). R^2^ denotes the square of the Pearson correlation coefficient.


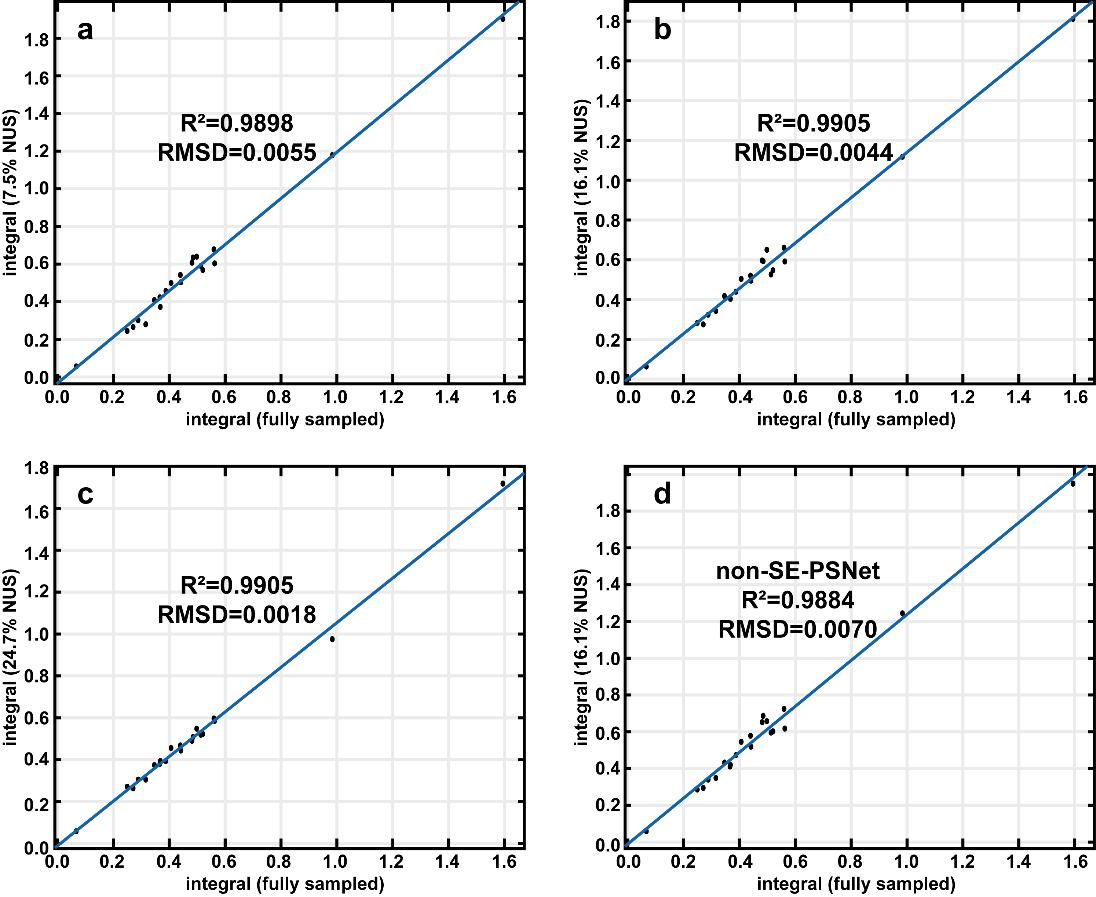


**Figure S6.** The quantitative evaluation using integrals between the fully sampled spectrum and the normalized SE-PSNet reconstruction of three NUS levels, namely 7.5% NUS (a), 16.1% NUS (b), 24.7% NUS (c) on quinine sample, in comparison with the non-SE-PSNet reconstruction for 16.1% NUS (d). The RMSD values are calculated between the reconstruction results and label regarding the peak regions.


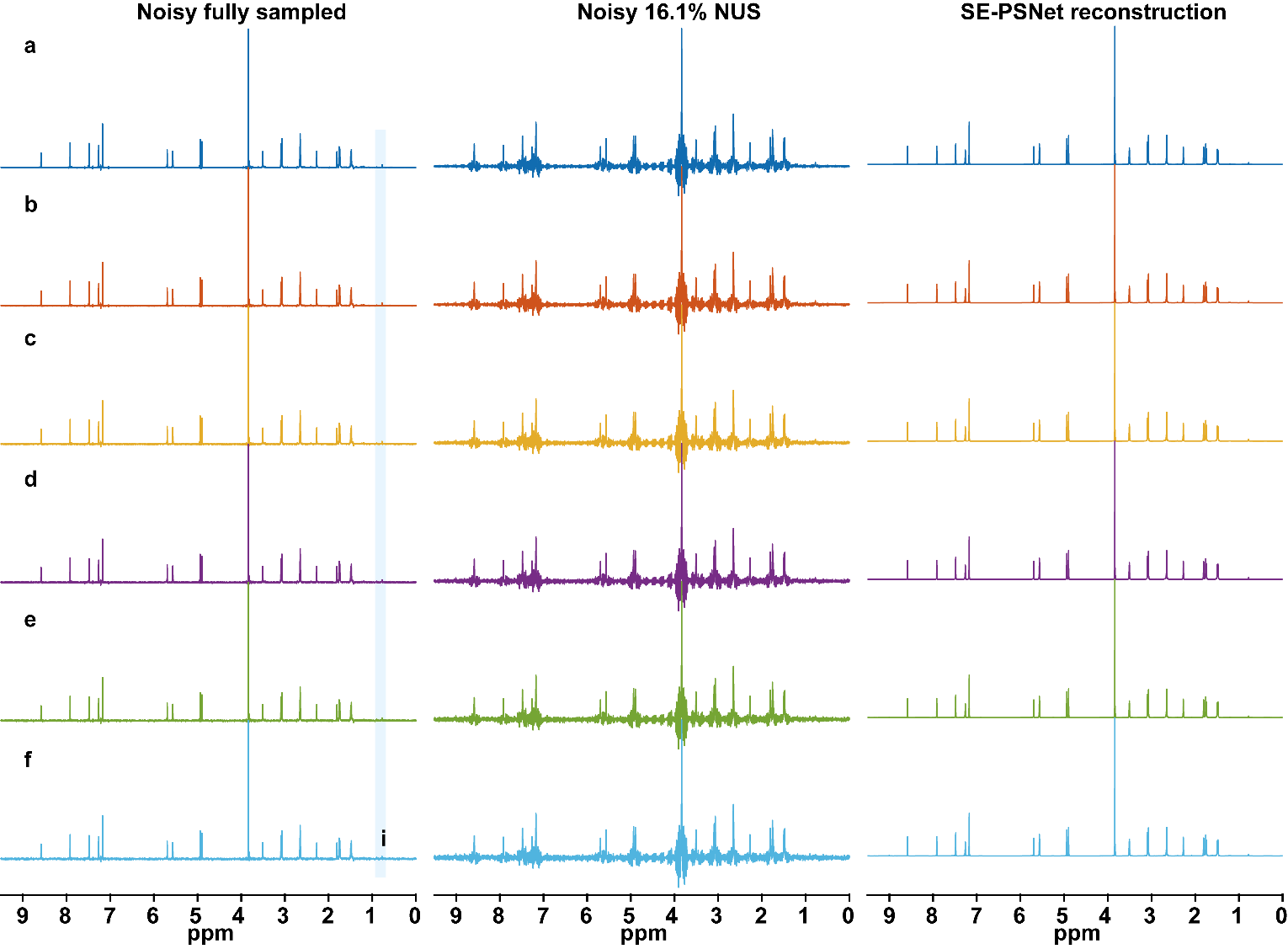


**Figure S7.** Pure shift reconstruction on quinine in the presence of noise. (a-f) The noisy fully sampled (left column), noisy 16.1% NUS inputs (middle column), and SE-PSNet reconstructions (right column) with different noise levels of *α*=1 (a), *α*=2 (b), *α*=3 (c), *α*=4 (d), *α*=5 (e), and *α*=6 (f). The identical SE-PSNet model to the Figure 3 and Figure 4, which was trained with the noise range *α* of the training datasets randomly set from 0 to 0.5, is adopted for desired pure shift reconstruction.


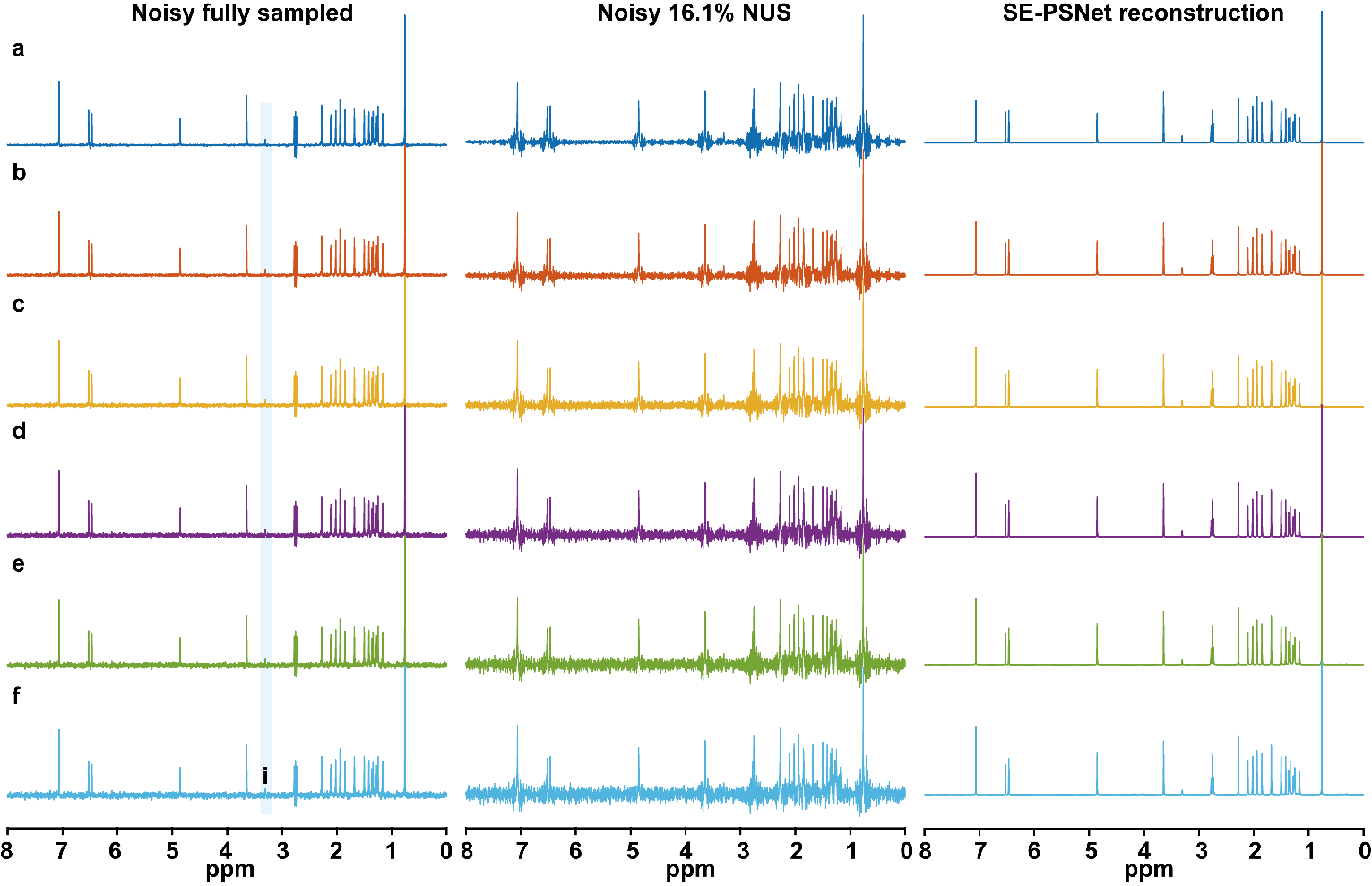


**Figure S8.** Pure shift reconstruction on β-estradiol in the presence of noise. (a-f) The noisy fully sampled (left column), noisy 16.1% NUS inputs (middle column), and SE-PSNet reconstructions (right column) with different noise levels of *α*=1 (a), *α*=2 (b), *α*=3 (c), *α*=4 (d), *α*=5 (e), and *α*=6 (f). 4000 synthetic samples are used to train the network model, and the noise range *α* of the training datasets were randomly set from 0 to 3.


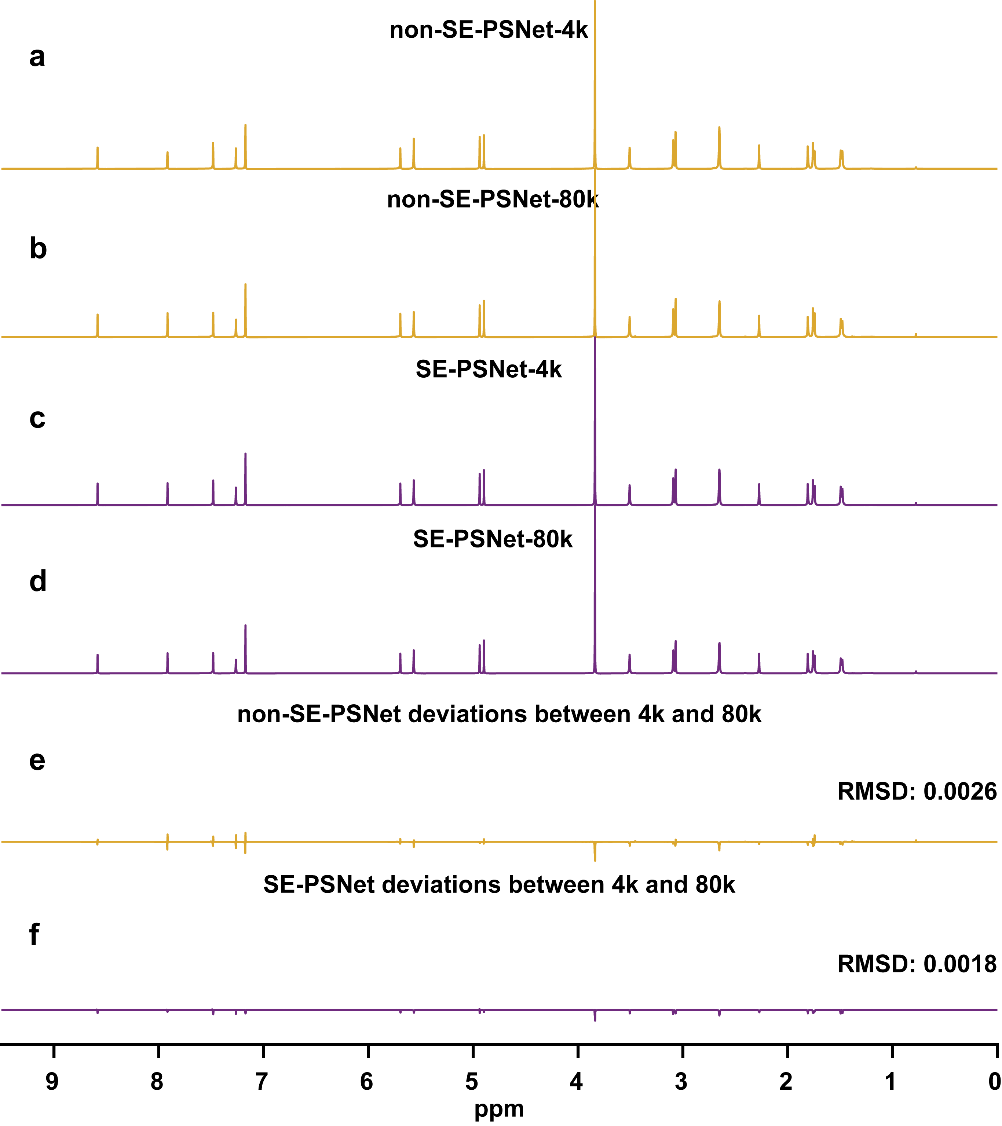


**Figure S9.** Pure shift reconstruction using the non-SE-PSNet and SE-PSNet models with the number of used training datasets of 4k and 80k. (a, b) The non-SE-PSNet reconstruction results with the number of used training datasets of 4k and 80k. (c, d) The SE-PSNet reconstruction results with the number of used training datasets of 4k and 80k. (e, f) The deviations of the non-SE-PSNet (e) and SE-PSNet (f) between the recontruction results with 4k and 80k synthetic data learning.


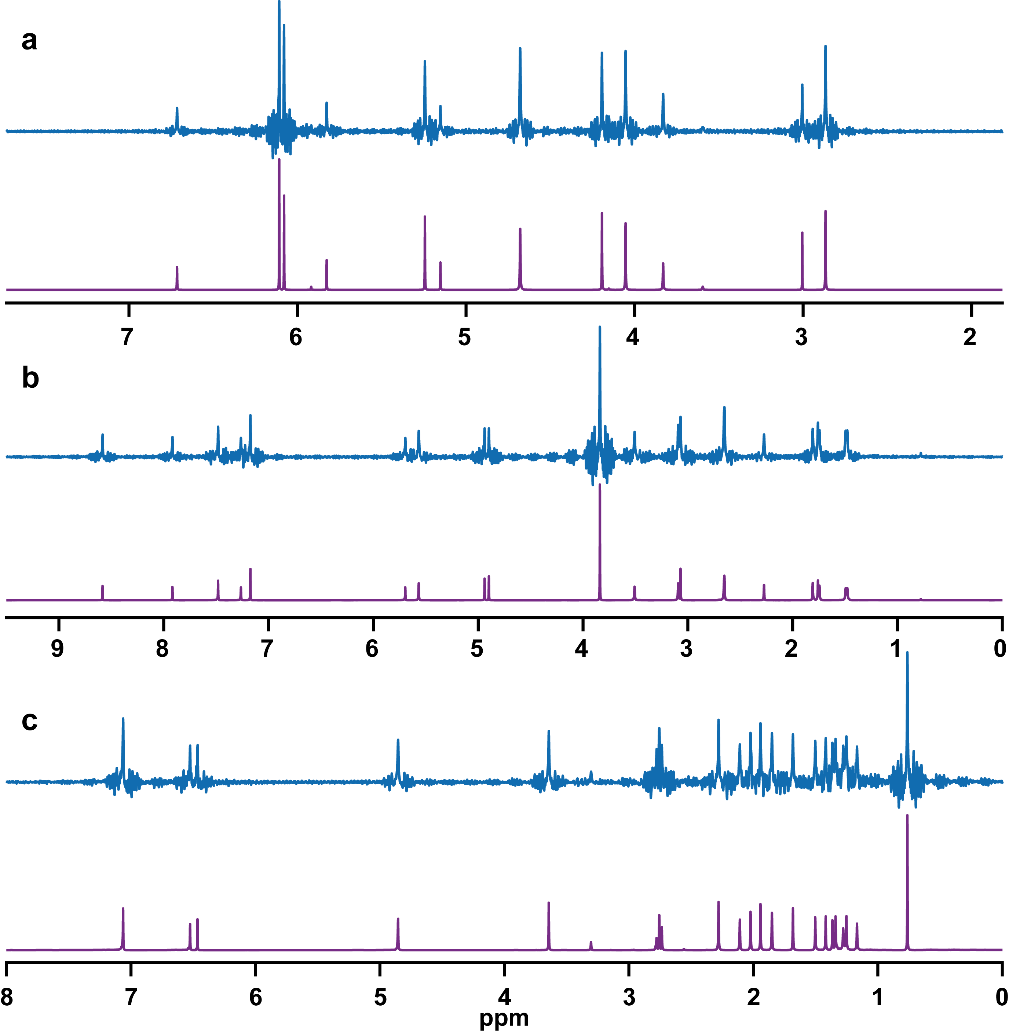


**Figure S10.** The 16.1% NUS inputs and related SE-PSNet reconstruction results adopting the Fourier transformation (FT) point of 16384 on the simulation sample (a), quinine (b), and β-estradiol (c).


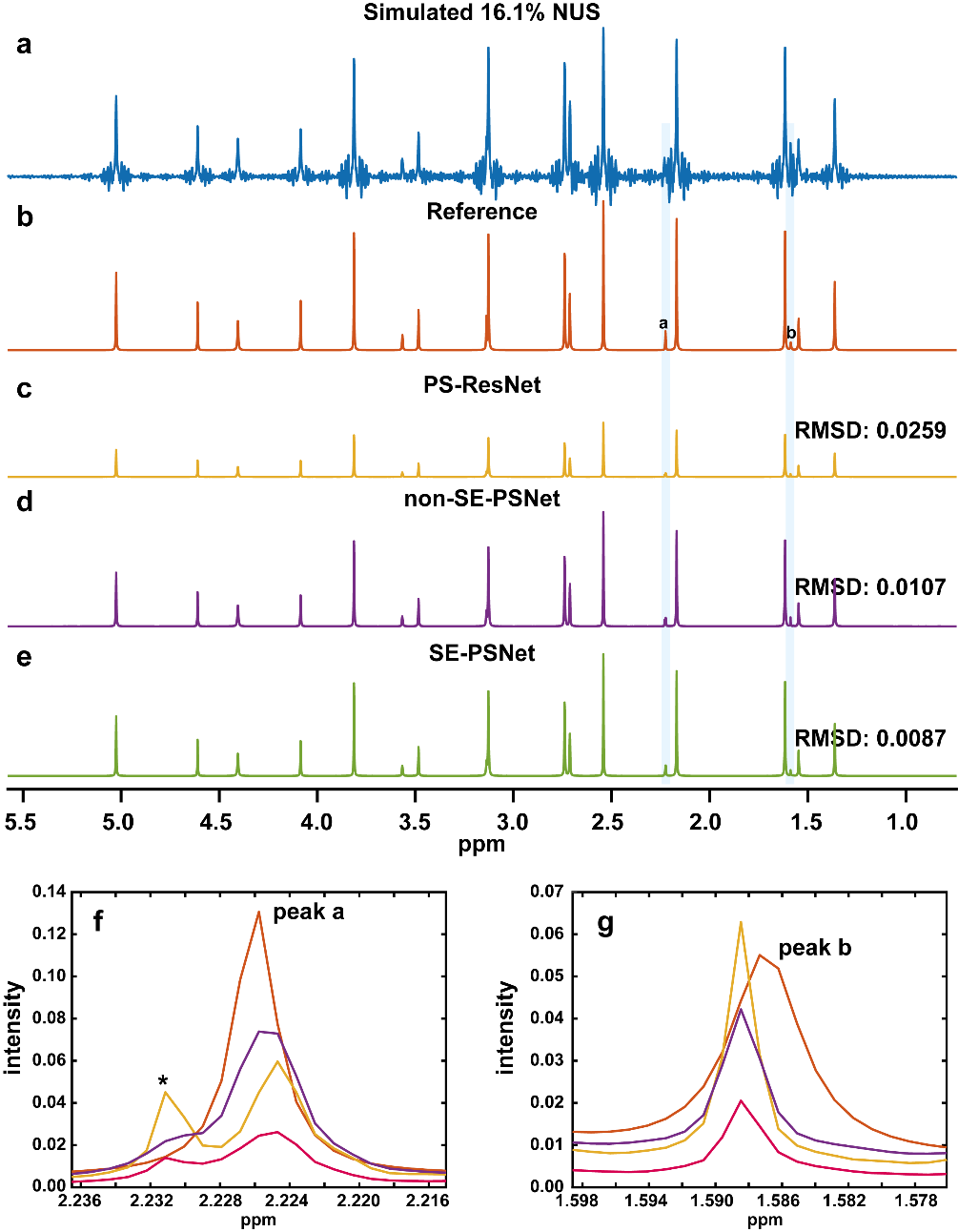


**Figure S11.** The detailed comparison of pure shift reconstruction among the PS-ResNet, non-SE-PSNet, and SE-PSNet. (a, b) 16.1% NUS pure shift spectrum and related ideal spectrum, which are the same as the Figure 2a and 2b. (c-e) Reconstruction results of the PS-ResNet (c), non-SE-PSNet (d), and SE-PSNet (e), which are directly obtained from the model outputs. (f, g) The zooms of peaks a and b marked by the arrows, in which the red, purple, and yellow denote the reference, SE-PSNet recontruction and non-SE-PSNet reconstruction results. The asterisks (*) denote the residual artifacts.


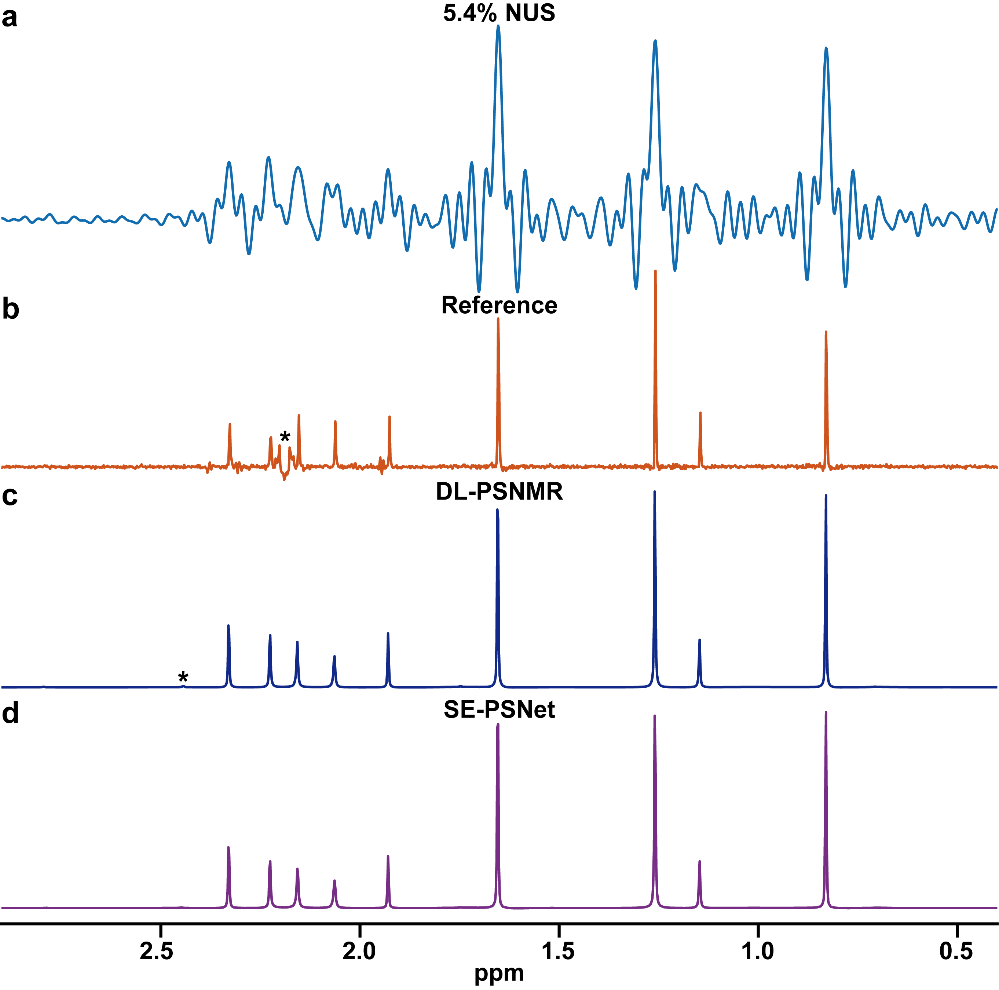


**Figure S12.** Pure shift reconstruction on 50mM α-pinene in CDCl_3_. (a) 5.4% NUS pure shift spectrum. (b) The fully sampled pure shift counterpart as a reference. (c, d) Reconstruction results of the DL-PSNMR (c) and SE-PSNet (d). The asterisks (*) in (b) and (c) denote the strong coupling artifacts and residual reconstruction artifacts for DL-PSNMR, respectively.


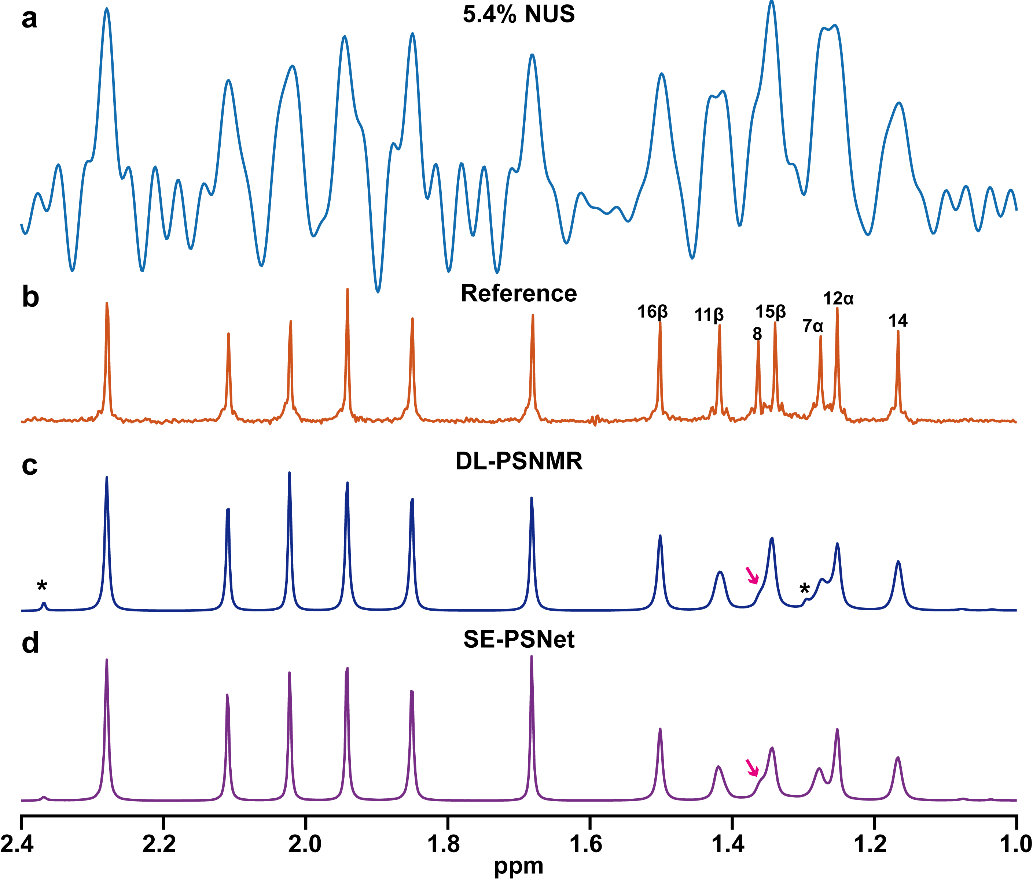


**Figure S13.** Pure shift reconstruction on β-estradiol. (a) 5.4% NUS pure shift spectrum. (b) The fully sampled pure shift counterpart as a reference. (c, d) Reconstruction results of the DL-PSNMR (c) and SE-PSNet (d). The asterisks (*) in (c) denote the residual reconstruction artifacts for DL-PSNMR, and the red arrow marks the reconstructed peak 8.


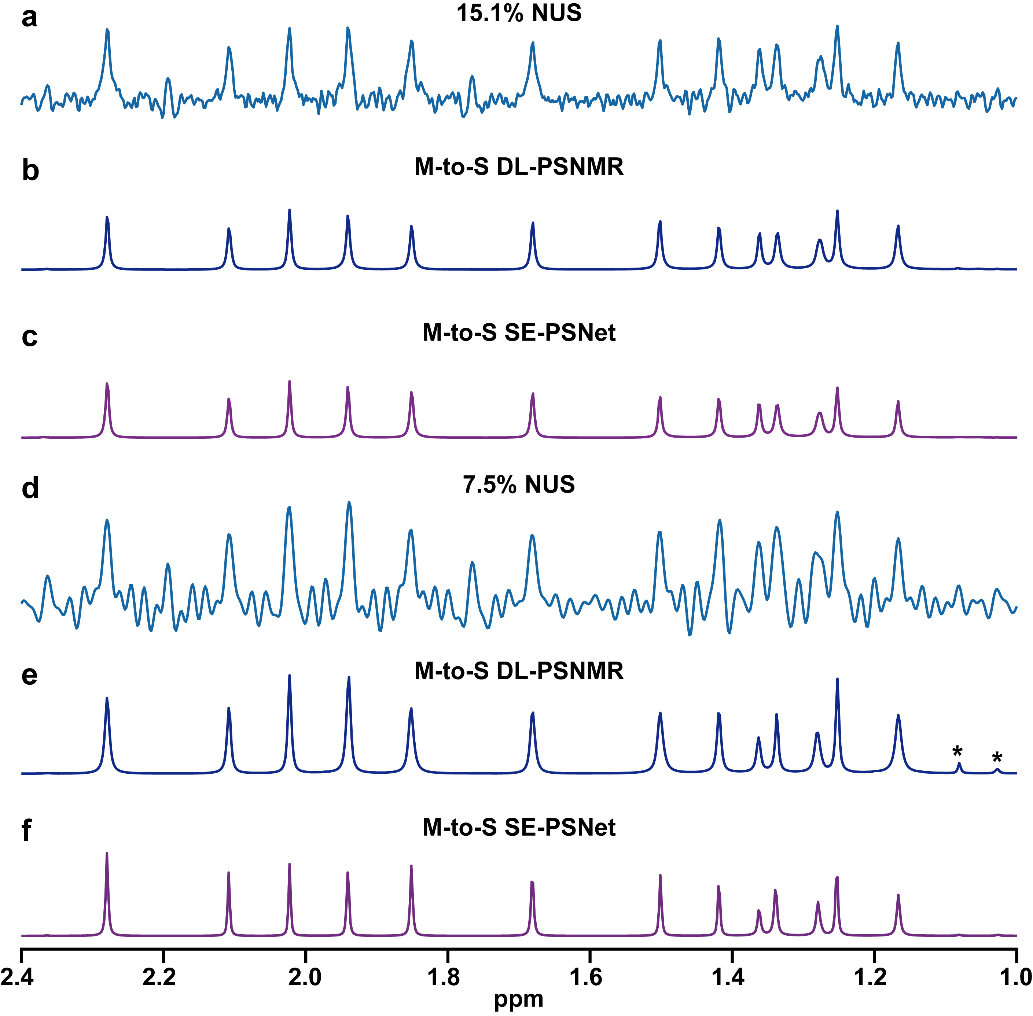


**Figure S14.** M-to-S pure shift NMR reconstruction on β-estradiol. (a) 15.1% NUS pure shift spectrum. (b, c) Reconstruction results on 15.1% NUS using the M-to-S DL PSNMR (b) and M-to-S SE-PSNet (c), in which 9 NUS inputs excluding the NUS levels of 15.1% and 7.5%, correspond to the same labels. (d) 7.5% NUS pure shift spectrum. (e, f) Reconstruction results on 15.1% NUS using the M-to-S DL PSNMR (e) and M-to-S SE-PSNet (f). The identical M-to-S models are adopted in (b) and (e), and in (c) and (f). The asterisks (*) in (e) denote the residual reconstruction artifacts for DL-PSNMR.

**References**

[1] K. Zangger, *Prog. Nucl. Magn. Reson. Spectrosc.* **2015**, *86-87*, 1-20.

[2] A. Shchukina, M. Kazmierczak, P. Kasprzak, M. Davy, G. R. Akien, C. P. Butts, K. Kazimierczuk, *Chem. Commun.* **2019**, *55*, 9563-9566.
